# Author Correction: Context-aware single-cell multiomics approach identifies cell-type-specific lung cancer susceptibility genes

**DOI:** 10.1038/s41467-024-54396-7

**Published:** 2024-11-18

**Authors:** Erping Long, Jinhu Yin, Ju Hye Shin, Yuyan Li, Bolun Li, Alexander Kane, Harsh Patel, Xinti Sun, Cong Wang, Thong Luong, Jun Xia, Younghun Han, Jinyoung Byun, Tongwu Zhang, Wei Zhao, Maria Teresa Landi, Nathaniel Rothman, Qing Lan, Yoon Soo Chang, Fulong Yu, Christopher I. Amos, Jianxin Shi, Jin Gu Lee, Eun Young Kim, Jiyeon Choi

**Affiliations:** 1 Division of Cancer Epidemiology and Genetics, National Cancer Institute, Bethesda, MD USA; 2 Department of Internal Medicine, Yonsei University College of Medicine, Seoul, Republic of Korea; 3 Institute of Basic Medical Sciences, Chinese Academy of Medical Sciences and Peking Union Medical College, Beijing, China; 4 Department of Biomedical Sciences, Creighton University, Omaha, NE USA; 5 Institute for Clinical and Translational Research, Baylor College of Medicine, Houston, TX USA; 6 Guangzhou National Laboratory, Guangzhou International Bio Island, Guangzhou, China; 7 Department of Thoracic and Cardiovascular Surgery, Yonsei University College of Medicine, Seoul, Republic of Korea; 8 Present Address: Institute of Basic Medical Sciences, Chinese Academy of Medical Sciences and Peking Union Medical College, Beijing, China

**Keywords:** Lung cancer, Gene expression profiling, Epigenomics, Risk factors, Cancer epidemiology

Correction to: *Nature Communications* 10.1038/s41467-024-52356-9, published online 12 September 2024

The original version of the Supplementary Information associated with this Article included an incorrect Supplementary Data 1-18 file, in which specific legends for each Supplementary Data table were missing. The HTML has been updated to include a corrected version of Supplementary Data 1-18.

