# Supplementary material for: Context-aware single-cell multiomics approach identifies cell-type-specific lung cancer susceptibility genes
Source: Nat Commun. 2024 Sep 12;15:7995. doi: 10.1038/s41467-024-52356-9 (PMC11392933; doi:10.1038/s41467-024-52356-9)
Supplement: Supplementary file 1 — Supplementary Information [file 41467_2024_52356_MOESM1_ESM.pdf]

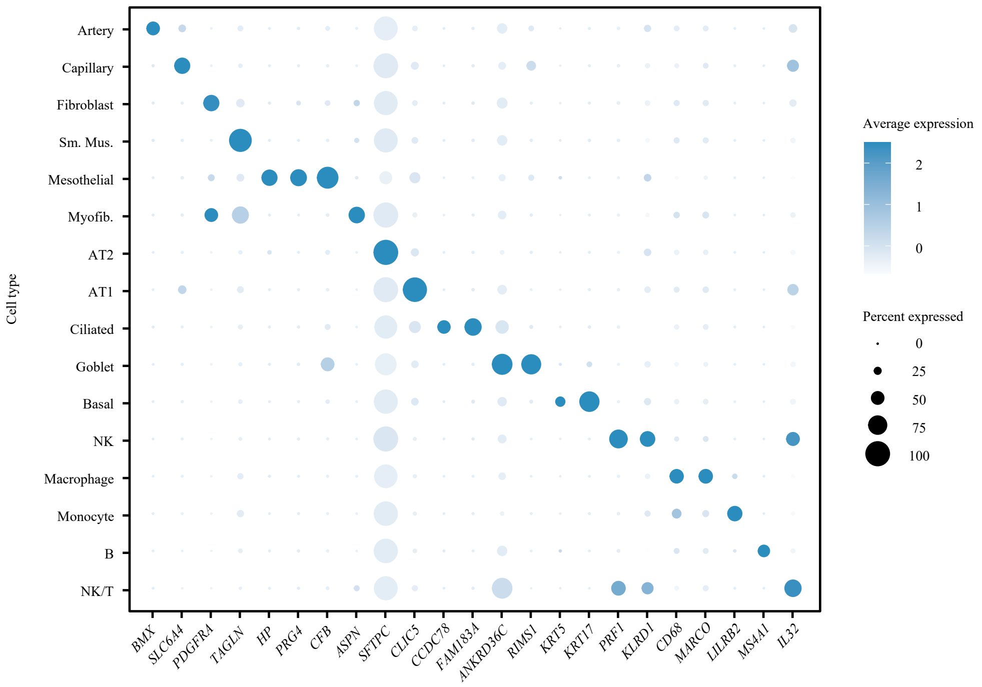

Figure S1. Additional canonical markers for cell-type annotation. Dot plot visualizing the normalized RNA expression of selected marker genes by cell type. The color and size of each dot correspond to the scaled average expression level and fraction of expressing cells, respectively. Source data are provided as a Source Data file.

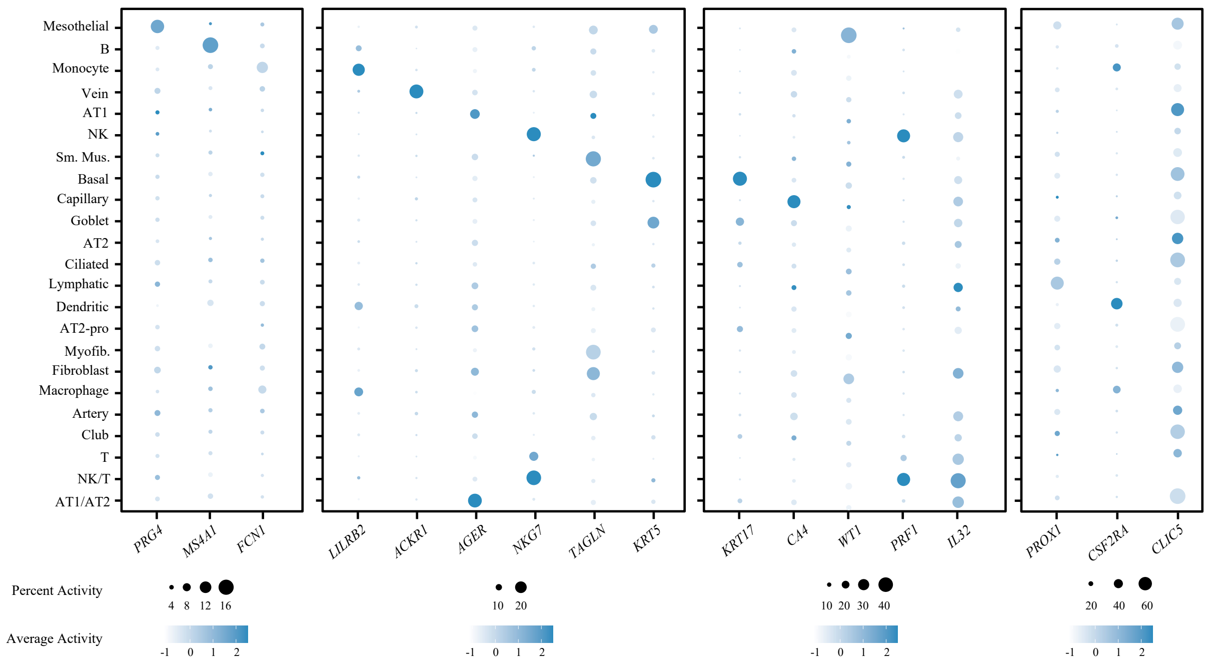

Figure S2. Seventeen canonical markers showing high consistency between gene expression and gene activity score. Dot plot visualizing the normalized gene activity score of canonical markers by cell type. The color and size of each dot correspond to the scaled average gene activity level and fraction of cells showing non-zero gene activity score, respectively. Source data are provided as a Source Data file.

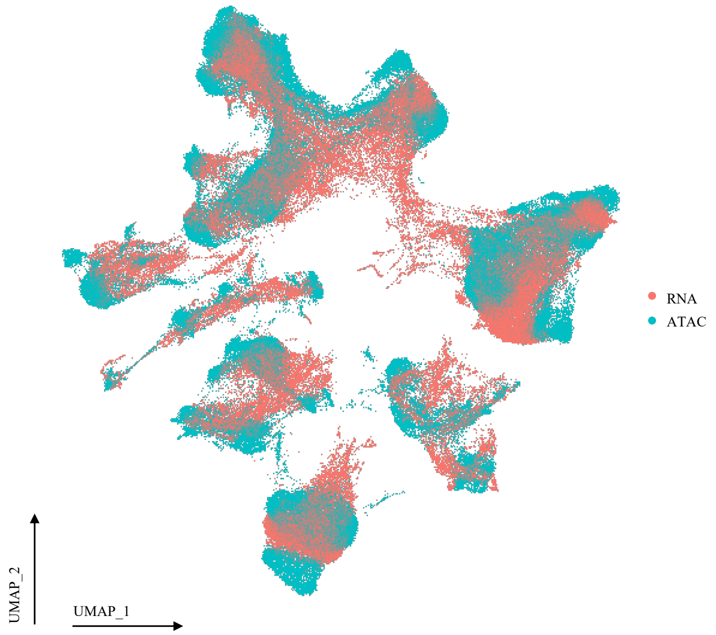

Figure S3. The visualization of UMAP co-embedding of RNA and ATAC modality. The UMAP co-embedding of the cells from snRNA-seq data and the snATAC–seq data on the same space color-coded by the two modalities (red, RNA; blue, ATAC).

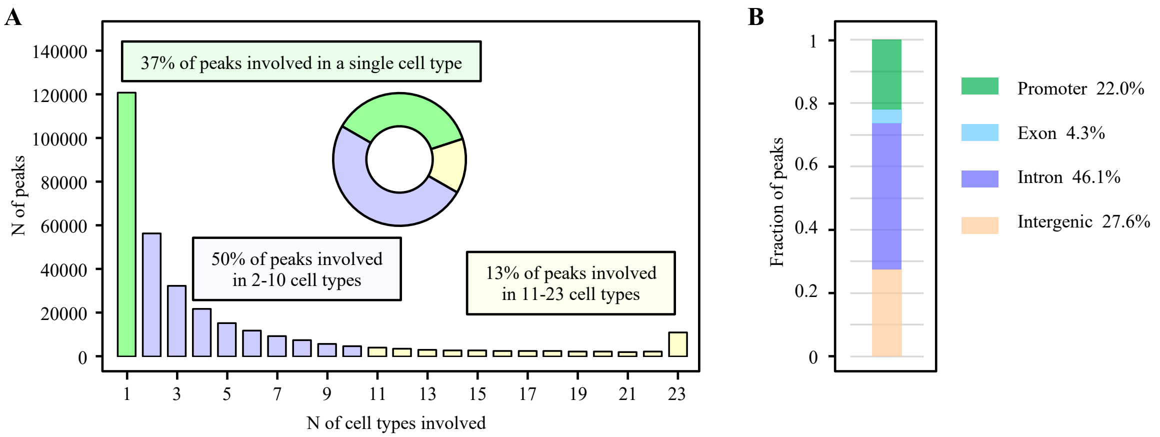

Figure S4. Accessible chromatin peaks identified from snATAC-seq data across different cell types. (A) Y-axis refers to number of peaks and X-axis refers to number of cell types involved. The piechart represents the proportion of peaks in single, 2-10, or 11-23 cell types. (B) Fraction of peaks were annotated into different categories (promoter, exon, intron, or intergenic). Source data are provided as a Source Data file.

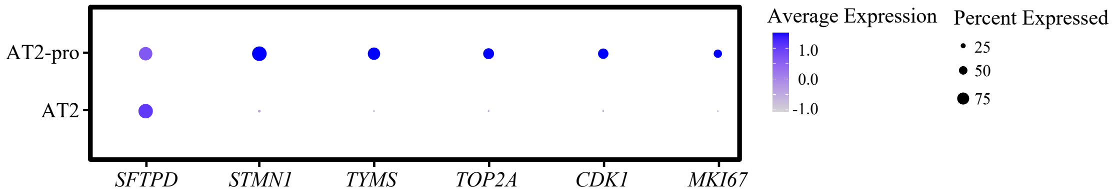

Figure S5. Marker gene expression of AT2-proliferating cells from HLCA dataset. Dot plot visualizing the normalized RNA expression of selected marker genes for AT2 and AT2-proliferation cells (AT2-pro) from HLCA dataset. A total of 61,429 AT2 cells and 976 AT-pro cells (based on the cell type annotation “ann_finest_level”) from 73 individuals were used for plotting. The color and size of each dot correspond to the scaled average expression level and fraction of expressing cells, respectively. Source data are provided as a Source Data file.

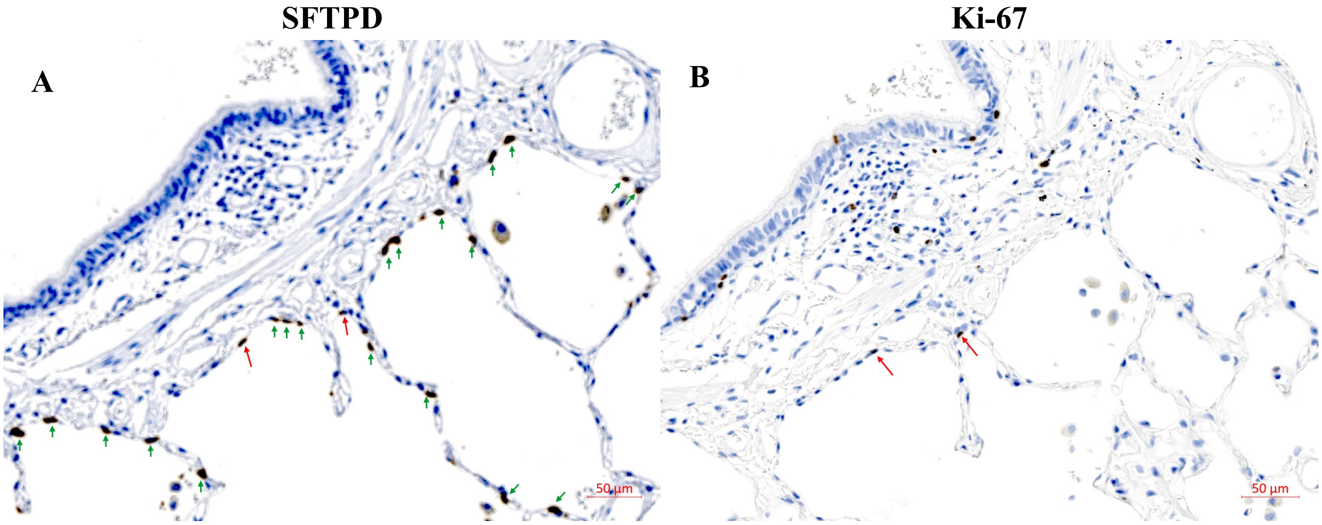

Figure S6. Detection of potential AT2-proliferating cells co-expressing SFTPD and Ki-67 in tumor-distant normal lung tissues. Representative images of immunohistochemical staining of (A) SFTPD and (B) Ki-67 in the adjacent sections of lung tissue from one of the female smokers sequenced in our dataset (labeled as FS4), where a subset of SFTPD-positive cells (suspected AT2 cells) was co-stained with Ki-67, indicating potential AT2-proliferating cells. Green arrows indicate likely AT2 cells, while red arrows indicate likely AT2-proliferating cells co-expressing SFTPD and Ki-67. The scale is shown in red at the bottom right-hand corner of each image. Positive antibody staining is shown in brown and nuclei staining in blue.

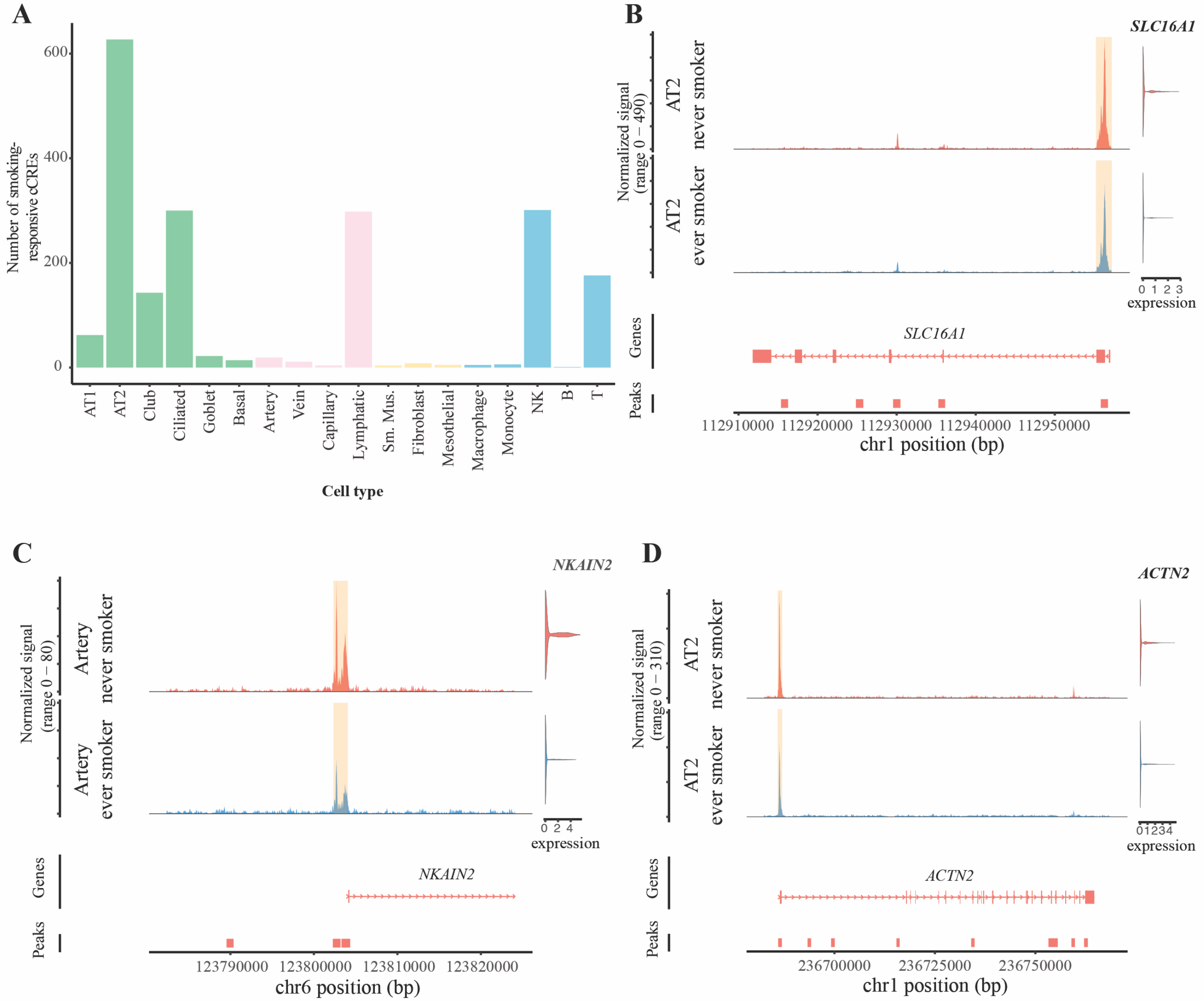

Figure S7. Smoking-responsive cCREs. (A) The number of significant smoking-responsive cCREs between ever- and never-smokers in each cell type. The bars are colored by cell categories (green: epithelial, red: endothelial, purple: stromal and blue: immune). (B-D) Peak signals of representative smoking-responsive cCREs and expression levels of their target genes (B: *SLC16A1*, C: *NKAIN2* and D: *ACTN2*) in the cell types that they were identified. The sequencing tracks representing chromatin accessibility of smoking-responsive cCREs are displayed. Each track represents the aggregated snATAC signal, normalized by the total number of reads in the regions. The regions of smoking-responsive cCREs are highlighted in beige. Gene expression levels are shown on the right side of normalized signal track. At the bottom, gene tracks show the direction of transcription by arrows and exons by boxes. The boxes in peak track represent cCREs called in the genomic region. Source data are provided as a Source Data file.

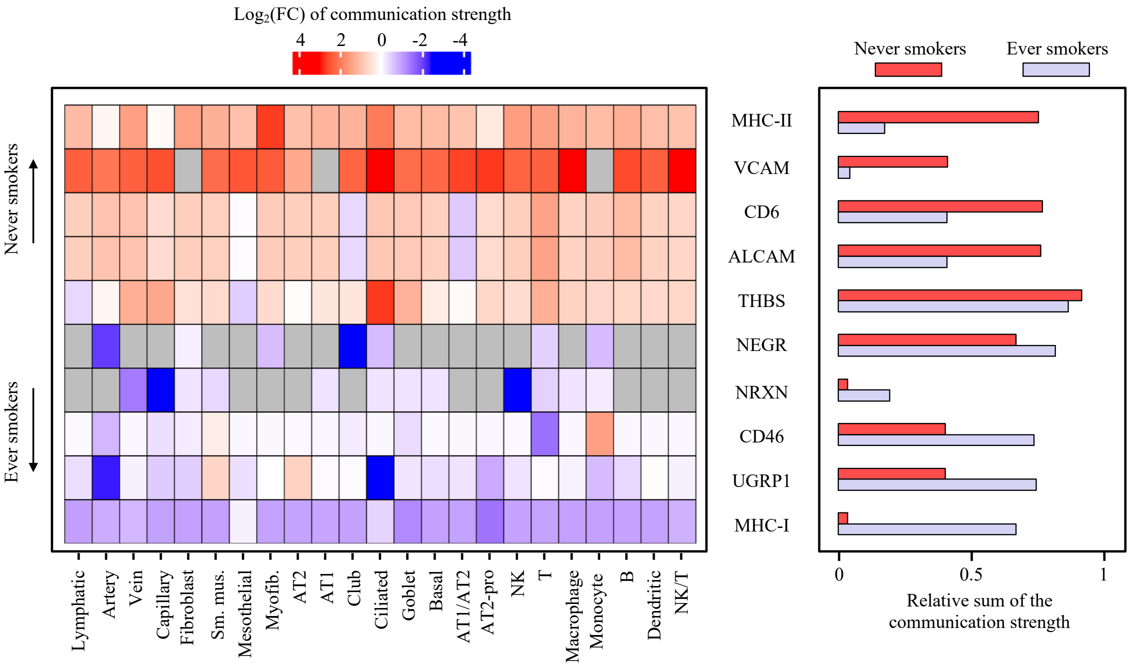

Figure S8. Differential intercellular communication based on smoking status. Heatmap of the top 5 elevated pathways in ever- (blue) and never-smokers (red) across cell types are shown on the left. Color indicates the log-transformed ratio of pathway-level communication strength between ever- and never-smokers (relative to never-smokers). Gray indicates that the pathway strength is 0 in the cell types of smokers and never smokers. The right part presents the summed communication strength of each pathway for ever- (blue) and never-smokers (red). Source data are provided as a Source Data file.

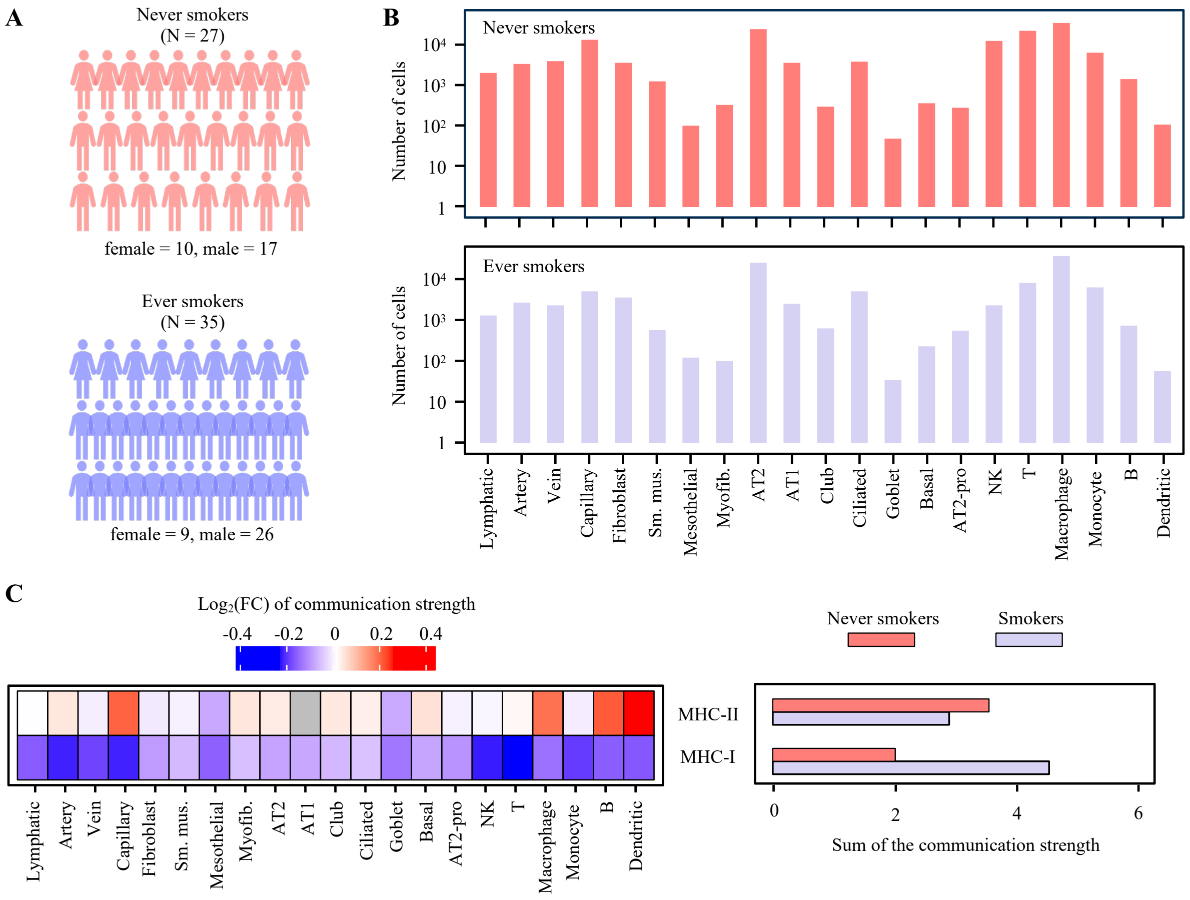

Figure S9. Validation of the MHC-I and MHC-II communication trend in the Human Lung Cell Atlas (HLCA) dataset. (A) The demographic information of ever- (blue) and never-smokers (red) from the HLCA core dataset that were included in the analysis. (B) The total number of cells across different cell types in ever- (blue) and never-smokers (red). (C) Heatmap of the communication strength of MHC-II and MHC-I pathways predicted by CellChat in ever- (blue) and never-smokers (red) across each cell type is shown on the left. Color indicates the $\log_2$-transformed ratio of pathway-level communication strength of never- over ever-smokers (positive value or red: stronger in never-smokers, negative value or blue: stronger in ever-smokers). Gray indicates that the pathway strength is 0 in the cell types of smokers and never-smokers. The right part presents the communication strength of each pathway for ever- (blue) and never-smokers (red) summed across all the cell types. Source data are provided as a Source Data file.

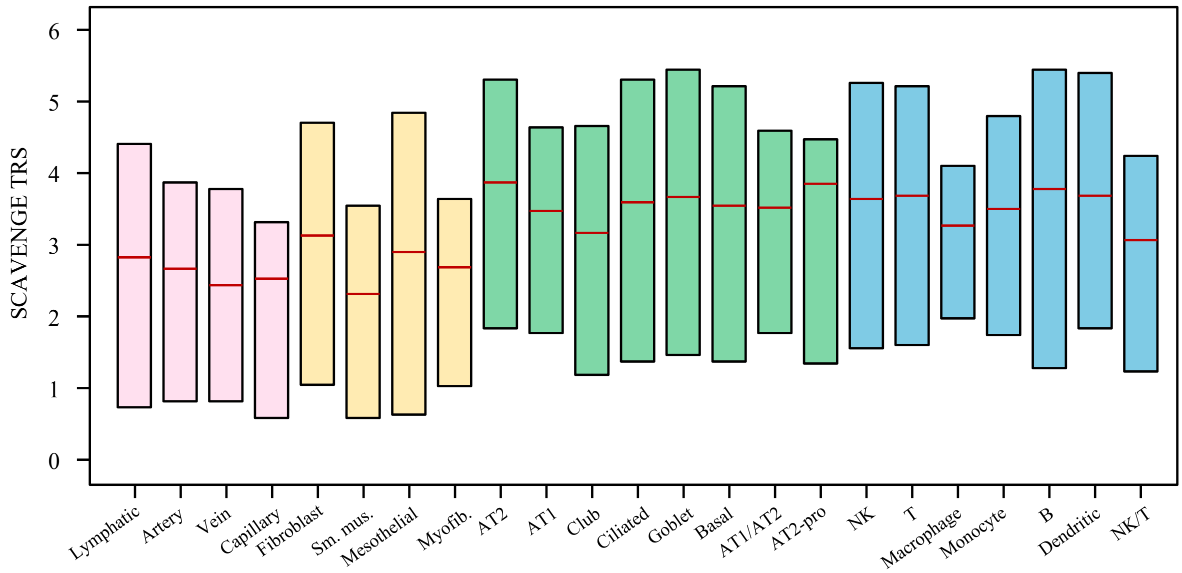

Figure S10. Trait relevance scores across cell types. The distribution of trait relevance scores (TRS) of lung cancer risk across cell types (endothelial in red, stromal in yellow, epithelial in green, and immune in blue) were presented as a boxplot. The red horizonal band shows the mean score, and the box indicates the middle 50% of cells. Source data are provided as a Source Data file.

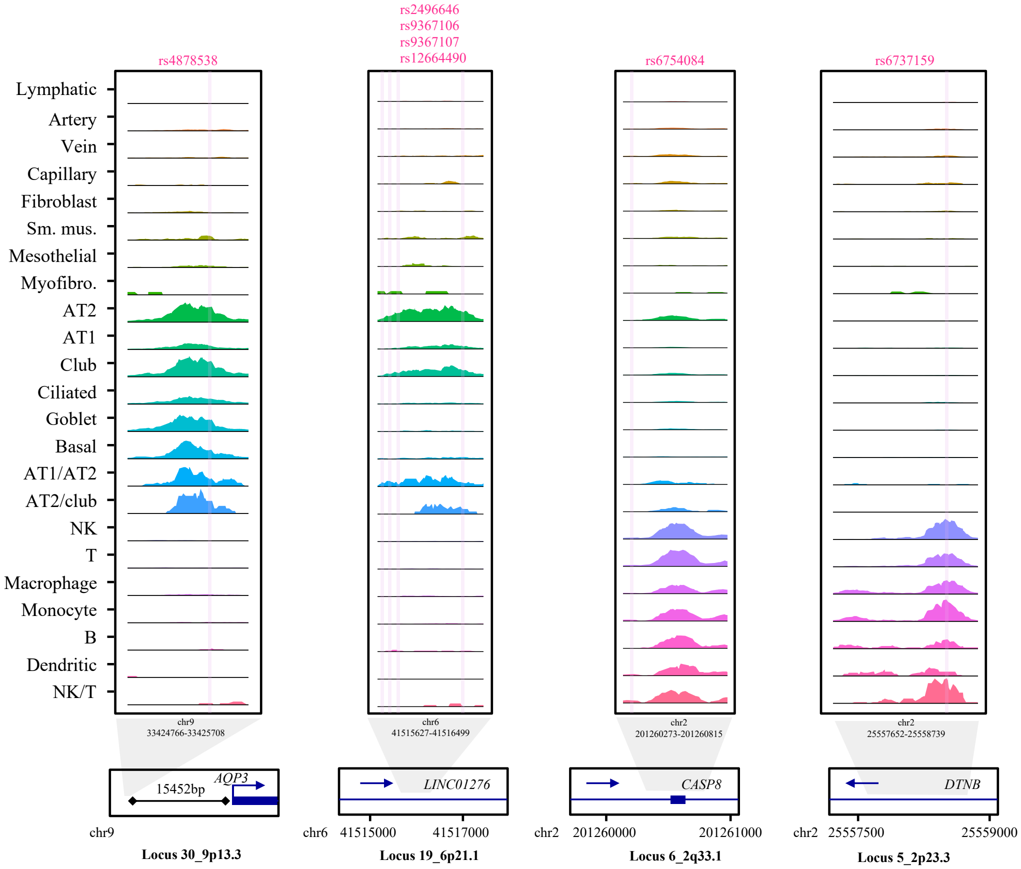

Figure S11. Epithelial or immune cell specific CCV-overlapping cCREs. The sequencing tracks representing chromatin accessibility of four different loci are displayed (locus IDs at the bottom). The rsIDs of CCVs are shown above the tracks and marked with vertical pink lines to indicate their genomic positions. Each track represents the aggregated snATAC signal of all cell types, normalized by the total number of reads in the regions (normalized values from left to right: 0-180, 0-180, 0-500, 0-240). Arrows depict the transcriptional directions of each gene.

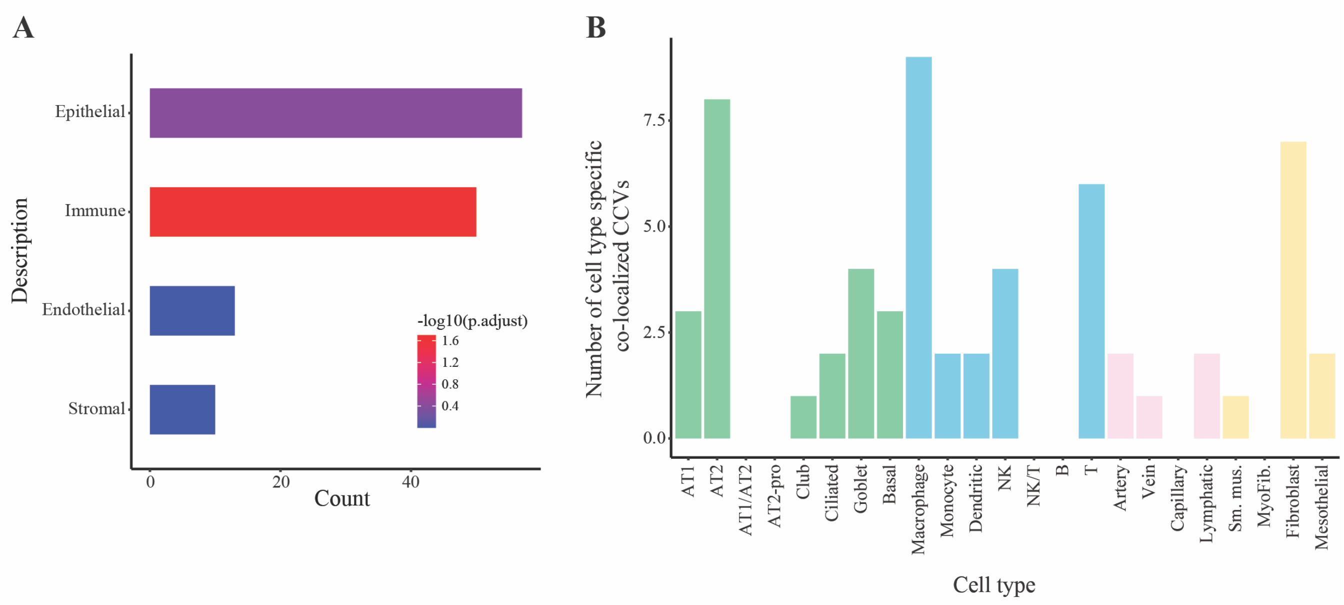

Figure S12. Enrichment and cell type specificity of CCV-colocalized cCREs. (A) The bar plot exhibits the number of cCREs co-localized with CCVs among total category-specific cCREs. The bars are colored by the -$\log_{10}$ transformed adjusted *P* values from hypergeometric test with Benjamini–Hochberg procedure as FDR correction, where immune cells show a significant enrichment (adjusted $P = 0.0198$). (B) The number of CCVs colocalized with cell-type specific cCREs in each cell type. The bars are colored by cell categories (blue: epithelial, purple: immune, green: endothelial, and yellow: stromal). Source data are provided as a Source Data file.

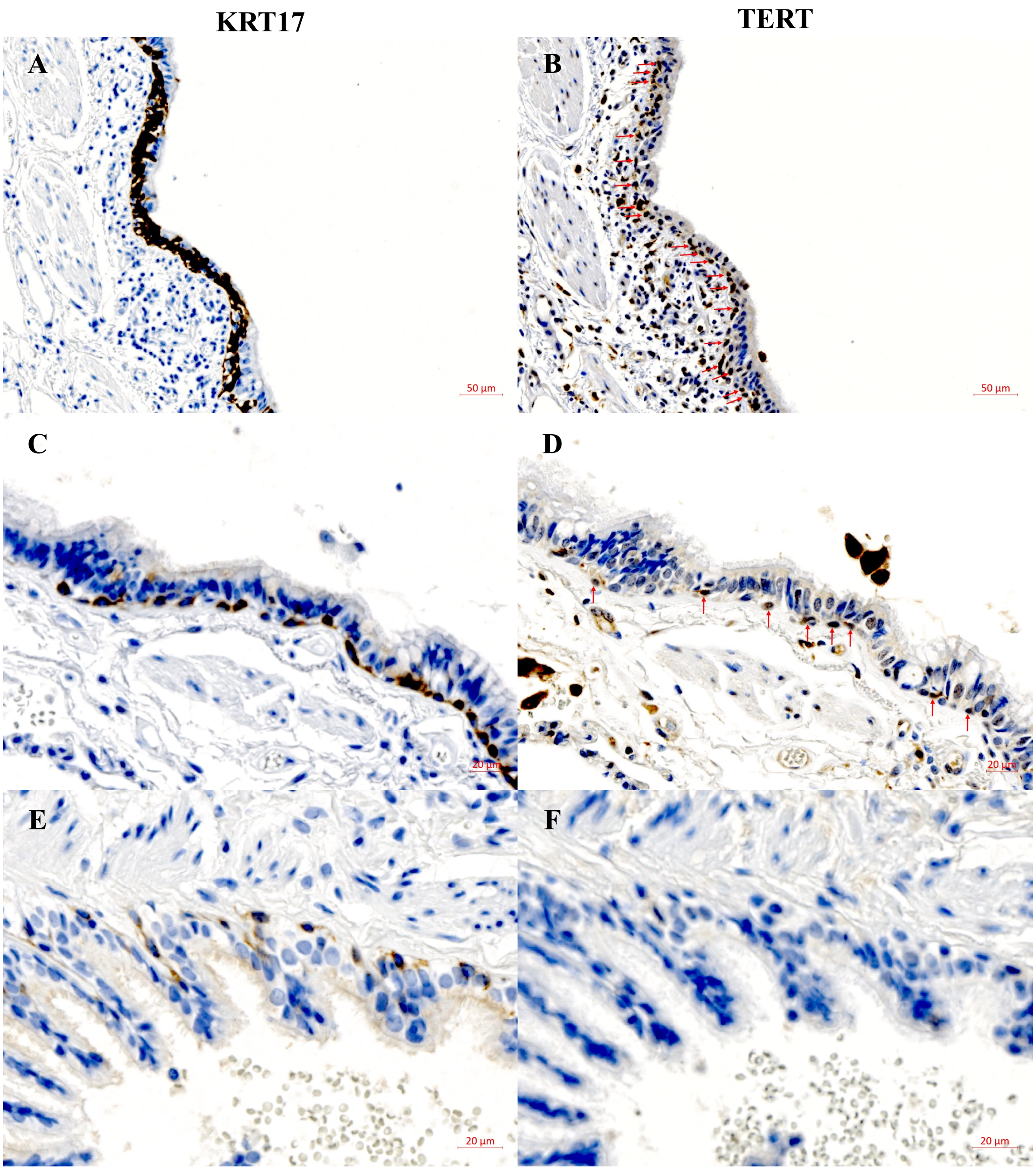

Figure S13. **Expression of KRT17 and TERT in tumor-distant normal lung tissues**. Representative images from immunohistochemical staining of KRT17 and TERT in the basal cells of normal lung bronchial epithelium. Red arrows indicate KRT17-positive basal cells with detectable TERT staining at a magnification of obj. x20 (B) or obj. x40 (D). The scale is shown in red at the bottom right-hand corner of each image. (A) Strong KRT17 expression in basal cells of lung tissue from a female smoker, FS4. (B) Moderate TERT expression in a subset of basal cells in an adjacent section from the same tissue. (C) Moderate KRT17 expression in basal cells of lung tissue from a male non-smoker, MN4. (D) Weak TERT expression in a subset of basal cells in an adjacent section from the same tissue. (E) Weak KRT17 expression in basal cells of lung tissue from a female non-smoker, FN3. (F) No detectable TERT expression in a subset of basal cells in an adjacent section from the same tissue. Positive antibody staining is shown in brown and nuclei staining in blue.

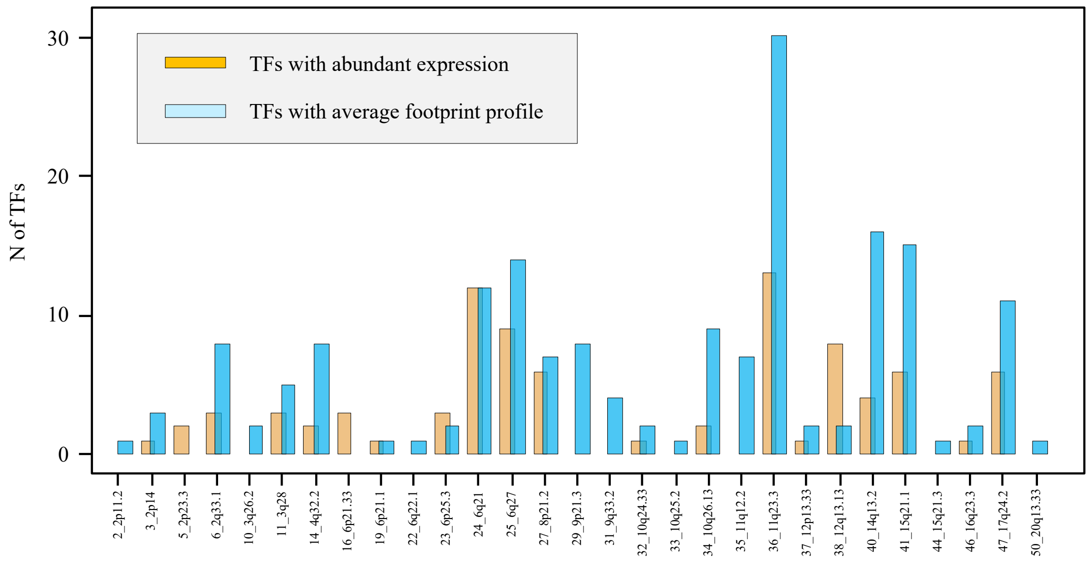

Figure S14. Distribution of the allelic transcription factors (TFs) across GWAS loci that were “abundantly expressed” or with a detectable average footprint profile. An “abundantly expressed” TF (yellow) was defined if it was expressed in >50% of the cells of a given cell type and the TF expression level in that cell type is above 75 percentile of the levels among all the predicted allelic TFs. TFs with a detectable average footprint profile (blue) were defined if the accessibility measured by Tn5 insertion in their motif-flanking regions was enriched over the background levels across the peaks in the genome in one or more cell type. Source data are provided as a Source Data file.

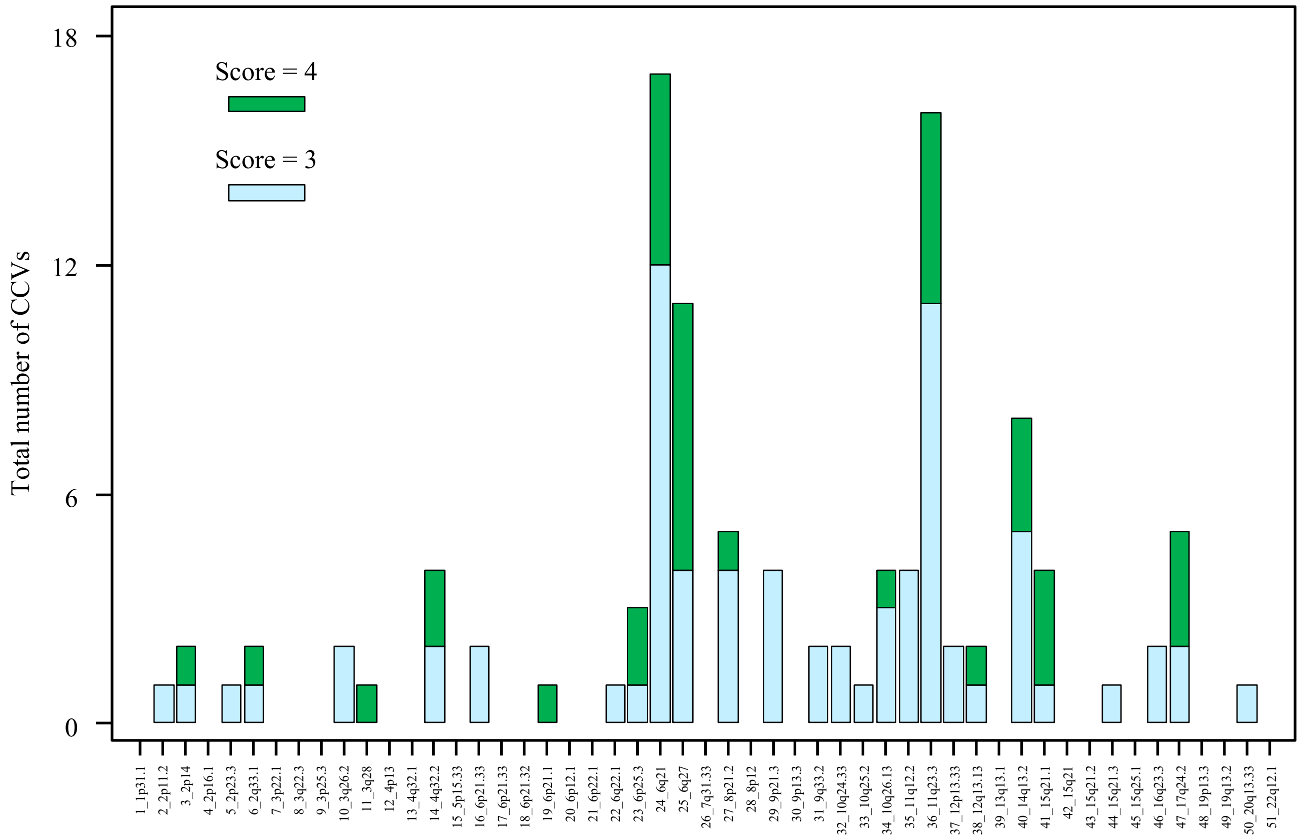

Figure S15. Total number of the CCVs scored 3 or 4 in each locus by TF expression and footprints. The cCRE-colocalized CCVs with an abundantly expressed TF or a TF footprint were assigned a score of 3 (blue). The cCRE-colocalized CCVs with an abundantly expressed TF and a TF footprint were assigned a score of 4 (green). Source data are provided as a Source Data file.

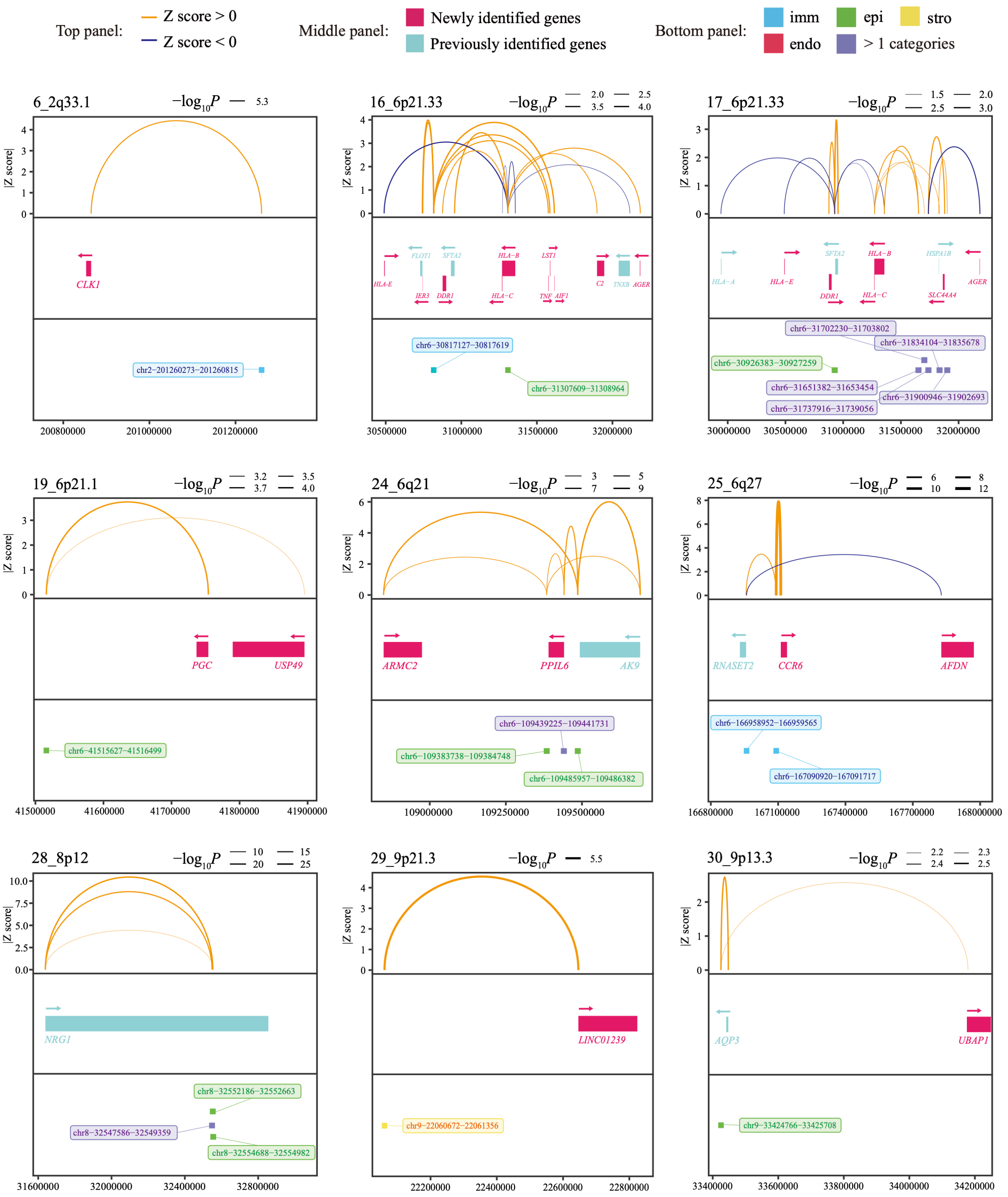
Top panel:
Z score > 0
Z score < 0
Middle panel:
Newly identified genes
Previously identified genes
Bottom panel:
imm
epi
stro
endo
> 1 categories
6_2q33.1
CLK1
chr2−201260273−201260815
16_6p21.33
chr6−30817127−30817619
chr6−31307609−31308964
17_6p21.33
chr6−31702230−31703802
chr6−31834104−31835678
chr6−30926383−30927259
chr6−31651382−31653454
chr6−31900946−31902693
chr6−31737916−31739056
19_6p21.1
PGC
USP49
chr6−41515627−41516499
24_6q21
ARMC2
PPIL6
AK9
chr6−109439225−109441731
chr6−109383738−109384748
chr6−109485957−109486382
25_6q27
RNASET2
CCR6
AFDN
chr6−166958952−166959565
chr6−167090920−167091717
28_8p12
NRG1
chr8−32552186−32552663
chr8−32547586−32549359
chr8−32554688−32554982
29_9p21.3
LINC01239
chr9−22060672−22061356
30_9p13.3
AQP3
UBAP1
chr9−33424766−33425708
|Z score|
−log10P

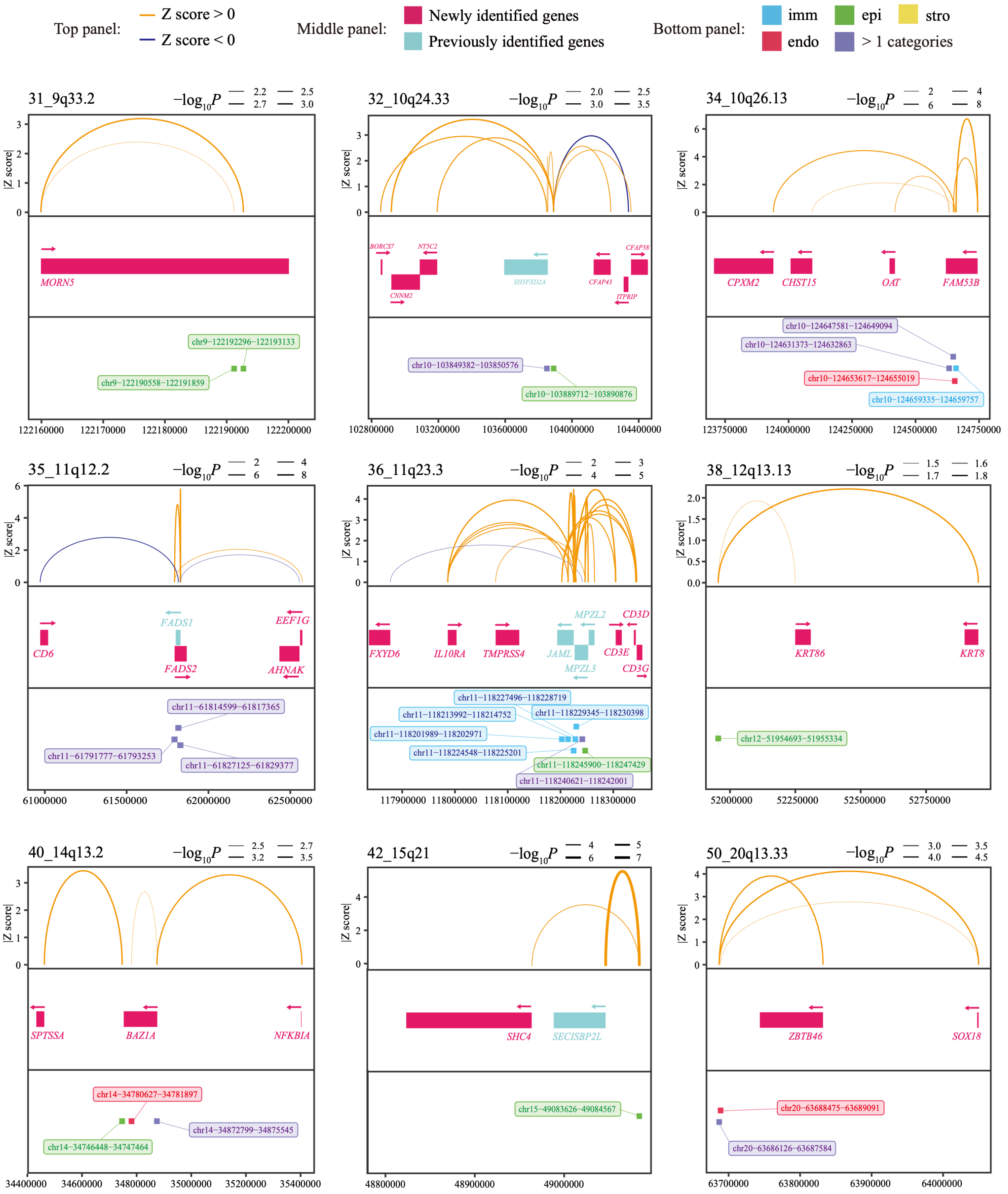

Figure S16. A summary of lung cancer susceptibility genes based on level-6 cCRE-gene linkage per locus. The eighteen loci with a level-6 linkage are shown. Each plot is divided into three panels: the top panel presents the absolute value of the cCRE-gene linkage Z score on the y-axis, with the Z score direction color-coded (yellow for positive and blue for negative correlation). The thickness of the loop refers to the -log$_{10}$(P) of the cCRE-gene linkage; the middle panel displays the level-6 genes (red for newly identified genes in this study, and green for previously identified genes). Arrow refers to the transcriptional direction. The bottom panel shows the cCREs with colors representing their cell type specificity (immune,

epithelial, endothelial, stromal, or multiple cell type categories). The width of cCRE is too small to visualize in scale, and thus we show cCREs in a fixed-sized square centered in their mid-point coordinate.

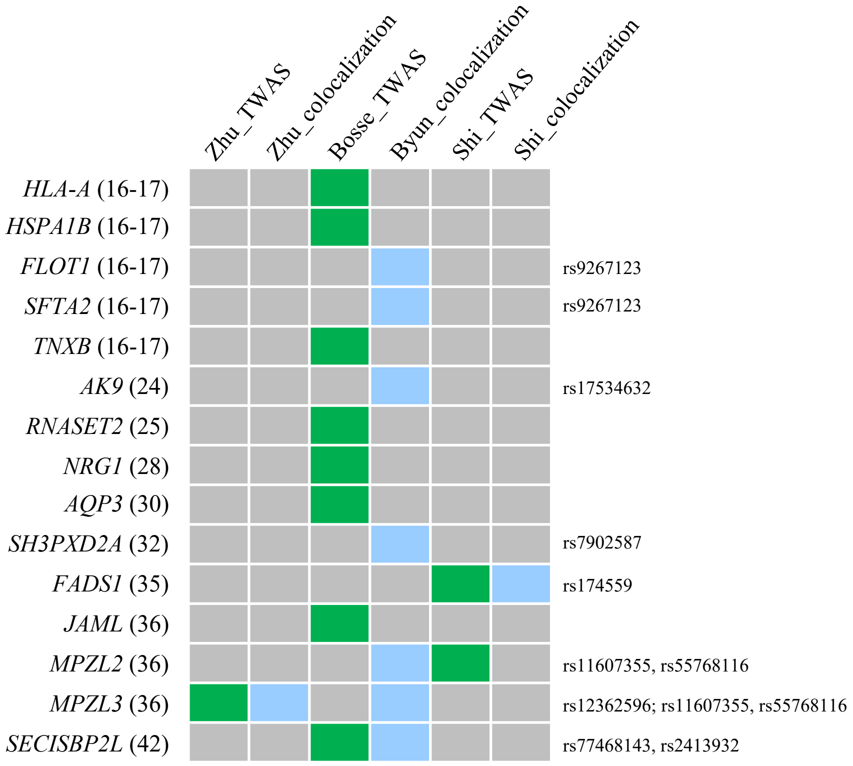

Figure S17. Loci with one or more level-6 genes that were identified by bulk-tissue eQTL-based colocalization or transcriptome-wide association studies (TWAS) from published studies. The loci serial IDs are in brackets after the gene names. Note that loci 16 and 17 are two MHC loci at 6p21.33 and share some target genes. Six criteria from four published studies are presented as columns (Zhu *et al*, PMID: 33909040, TWAS and colocalization; Bosse *et al*, PMID: 31696517, TWAS; Byun *et al*, PMID: 35915169, colocalization; Shi *et al*, PMID: 37236969, TWAS and colocalization). TWAS (green) and colocalization (blue) hits are color-coded. The corresponding variants prioritized by colocalization are presented on the right side of the columns. Variants prioritized by different studies are separated by semicolons.

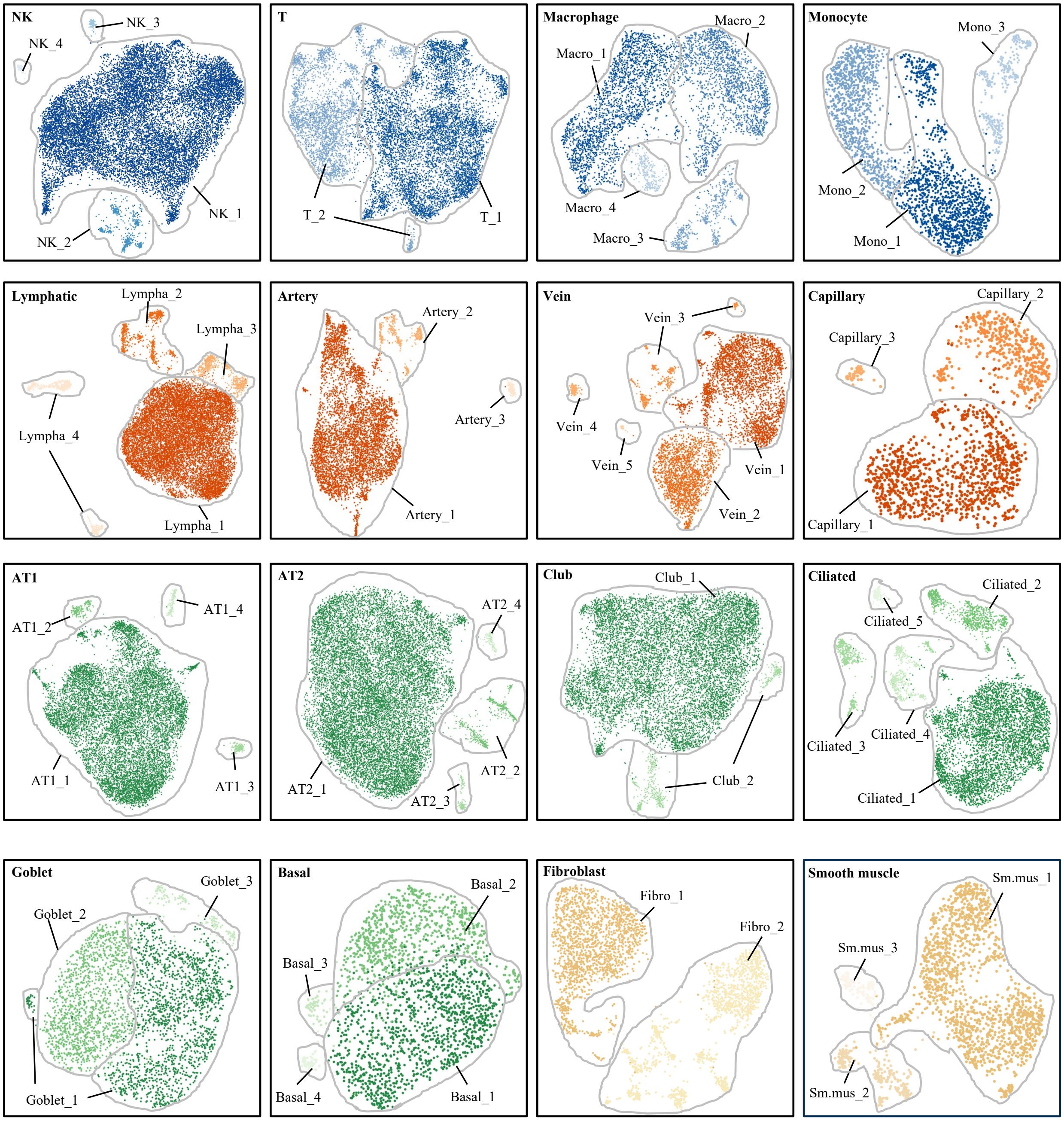

Figure S18. The Weighted Nearest Neighbor (WNN) dimension visualizations of subpopulation in each cell type. The visualizations of the cell subpopulations are displayed and marked with serial numbers followed by the cell type name. The X- and Y-axis represents the first and second dimension of WNN, respectively. Cell types in each panel are ordered by the category with different color scheme (immune: blue, endothelial: red, epithelial: green, and stromal: yellow).

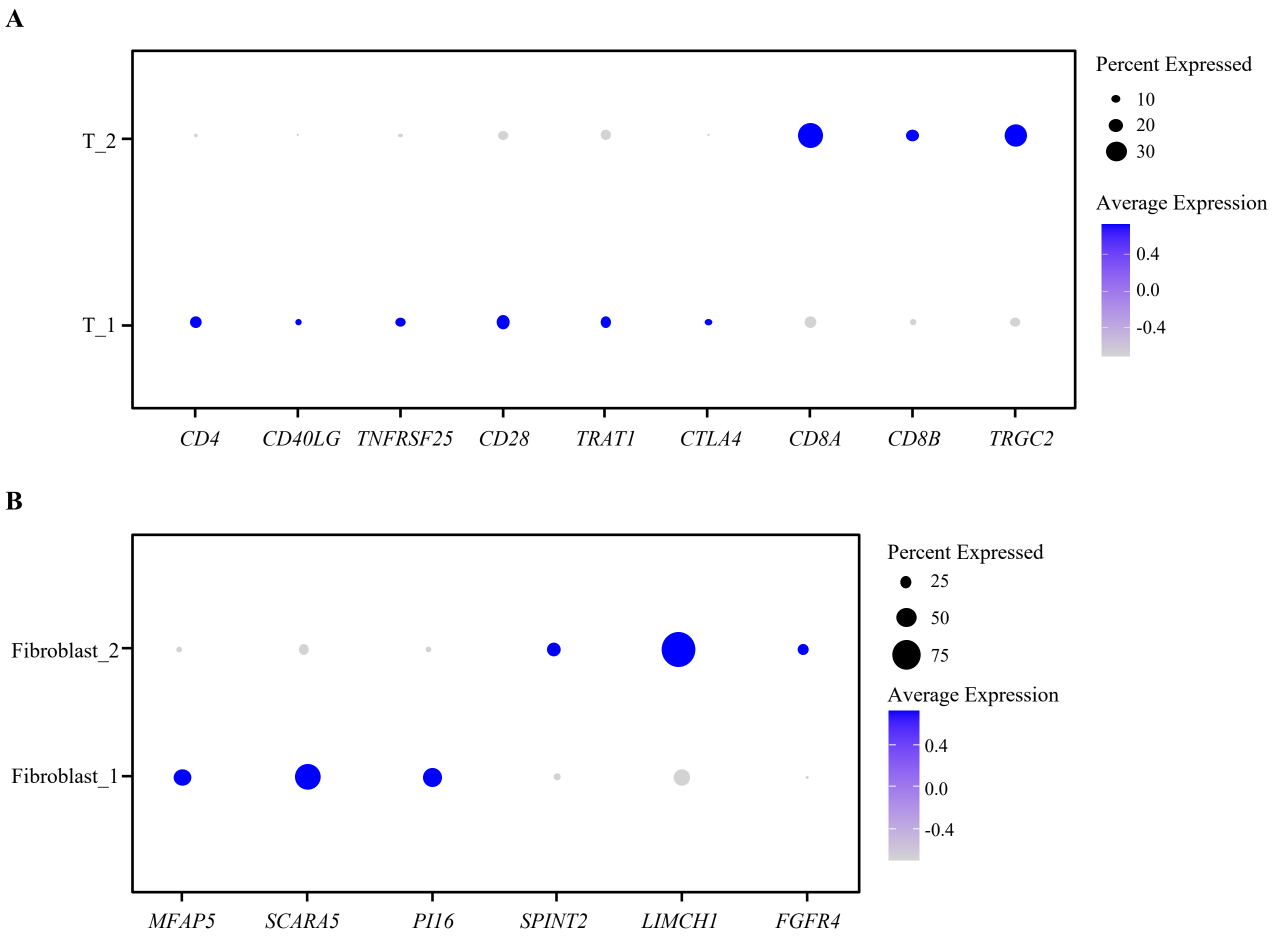

Figure S19. Representative subpopulations expressing known marker genes. Dot plot visualizing the normalized RNA expression of known marker genes for CD4 (*CD4*, *CD40LG*, *TNFRSF25*, *CD28*, *TRAT1*, and *CTLA4*) and CD8 (*CD8A*, *CD8B*, and *TRGC2*) T-cell subpopulations (A) and for adventitial (*MFAP5*, *SCARA5*, and *PI16*) and alveolar (*SPINT2*, *LIMCH1*, and *FGFR4*) fibroblasts subpopulations (B). The color and size of each dot correspond to the scaled average expression level and fraction of expressing cells, respectively. Source data are provided as a Source Data file.

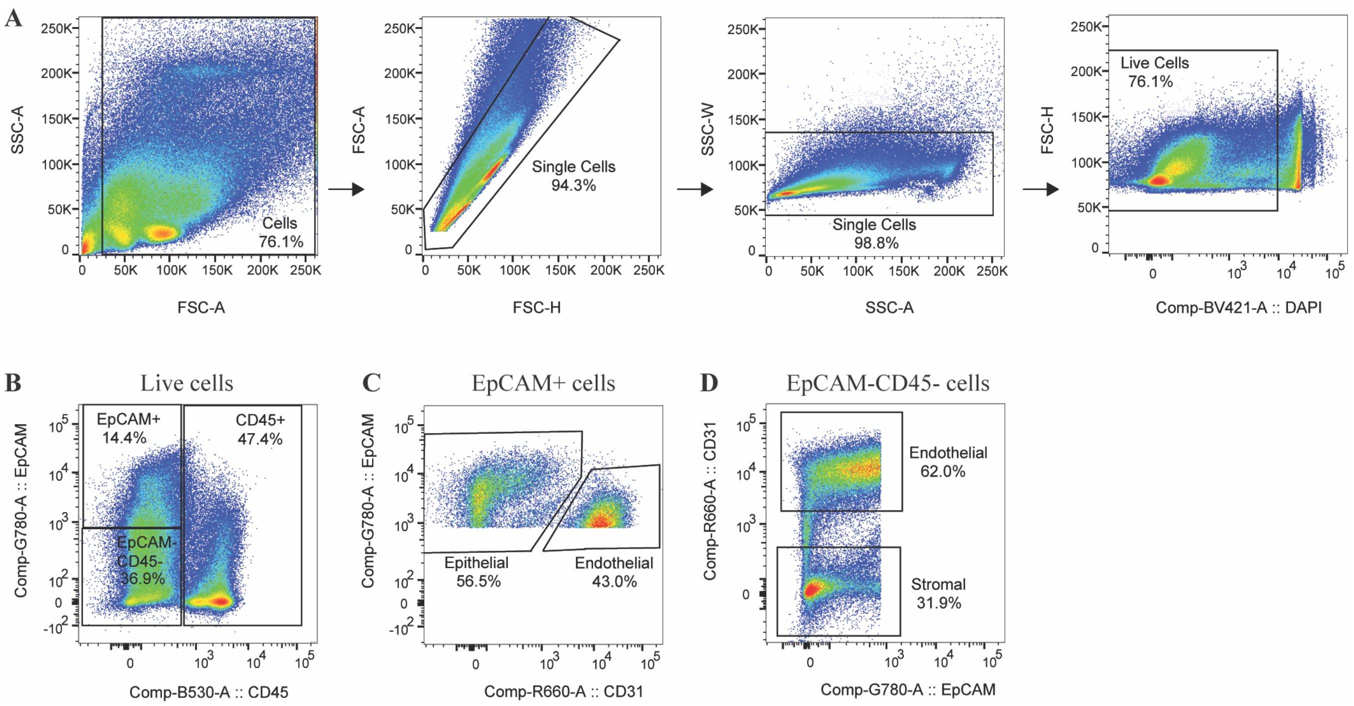

Figure S20. FACS gating strategies for dissociated human lung cells. (A) Sequential FACS data and sorting gates for dissociated human lung cells from a representative sample, MS3. Four steps of gating to sequentially isolate dissociated, single (second and third steps for forward scatter, FSC, and side scatter, SSC, respectively), and live cells are shown. The gating and the resulting percentage of sorted cells of each step are indicated in solid black lines. (B) From the live cells, epithelial cells ($EpCAM^+CD45^-$, "EpCam+"), immune cells ($CD45^+EpCAM^-$, "CD45+"), and stromal/endothelial ($EpCAM^-CD45^-$, "EpCAM-CD45-") were sorted based on the indicated gating and subsequently mixed 6:3:1 prior to nuclei isolation. (C) Subgating of endothelial ($CD31^+$) and epithelial ($EpCAM^+$) cells from the sorted "EpCam+" cells in B. (D) Subgating of endothelial ($CD31^+$) and stomal ($CD31^-$) cells from the sorted "EpCAM-CD45-" cells in B. Resulting percentages of the four cell type categories after subgating were used to calculate the cell category proportions before and after balancing as shown in **Figure 1F**.

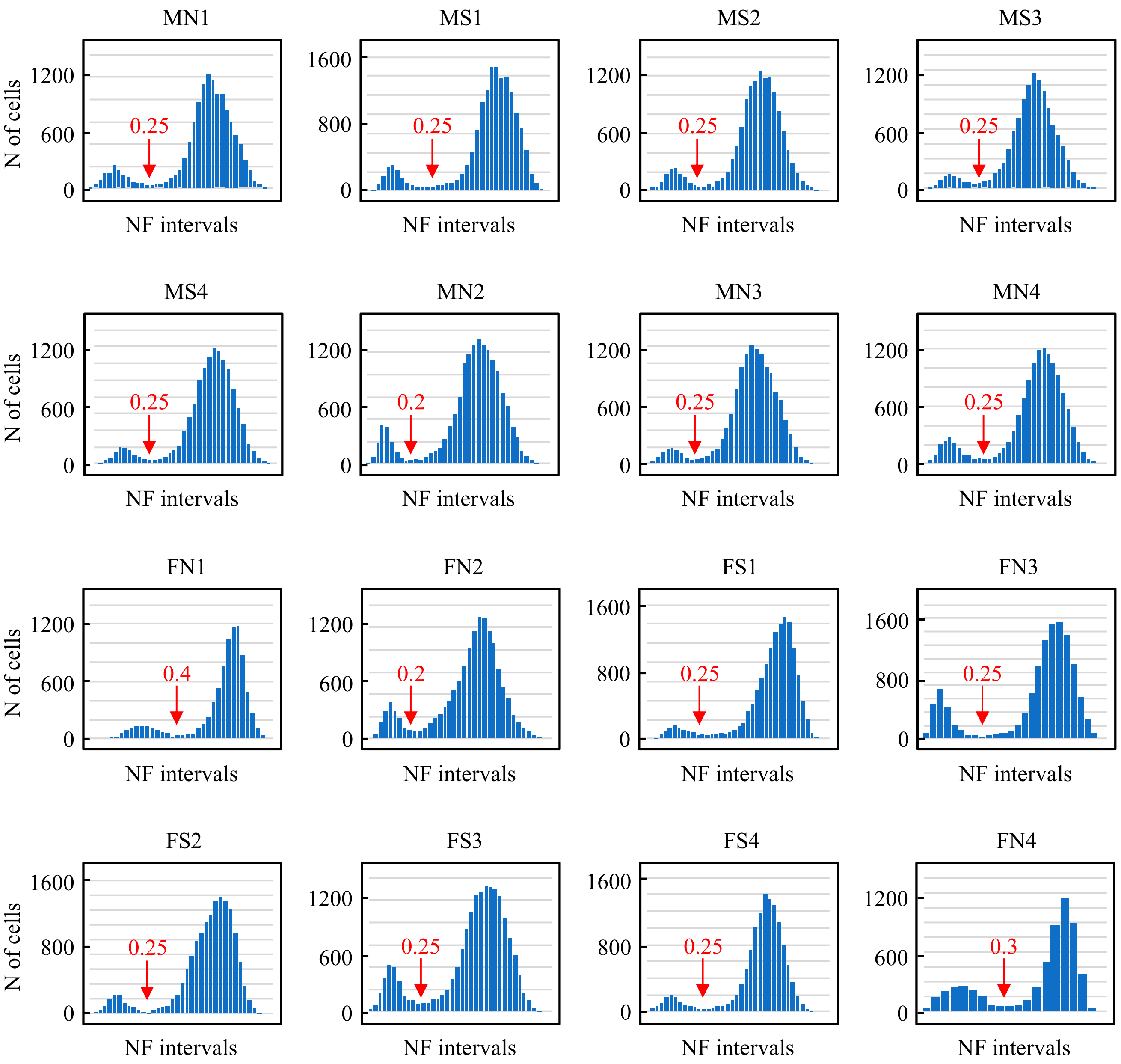

Figure S21. Determination of the cutoff in defining the empty droplet using DropletQC. The nuclear fraction (NF) of each cell is calculated by DropletQC. Empty droplets were identified by visualizing the density of nuclear fraction and setting the cutoff according to the "peak" in low-nuclear-fraction droplets.

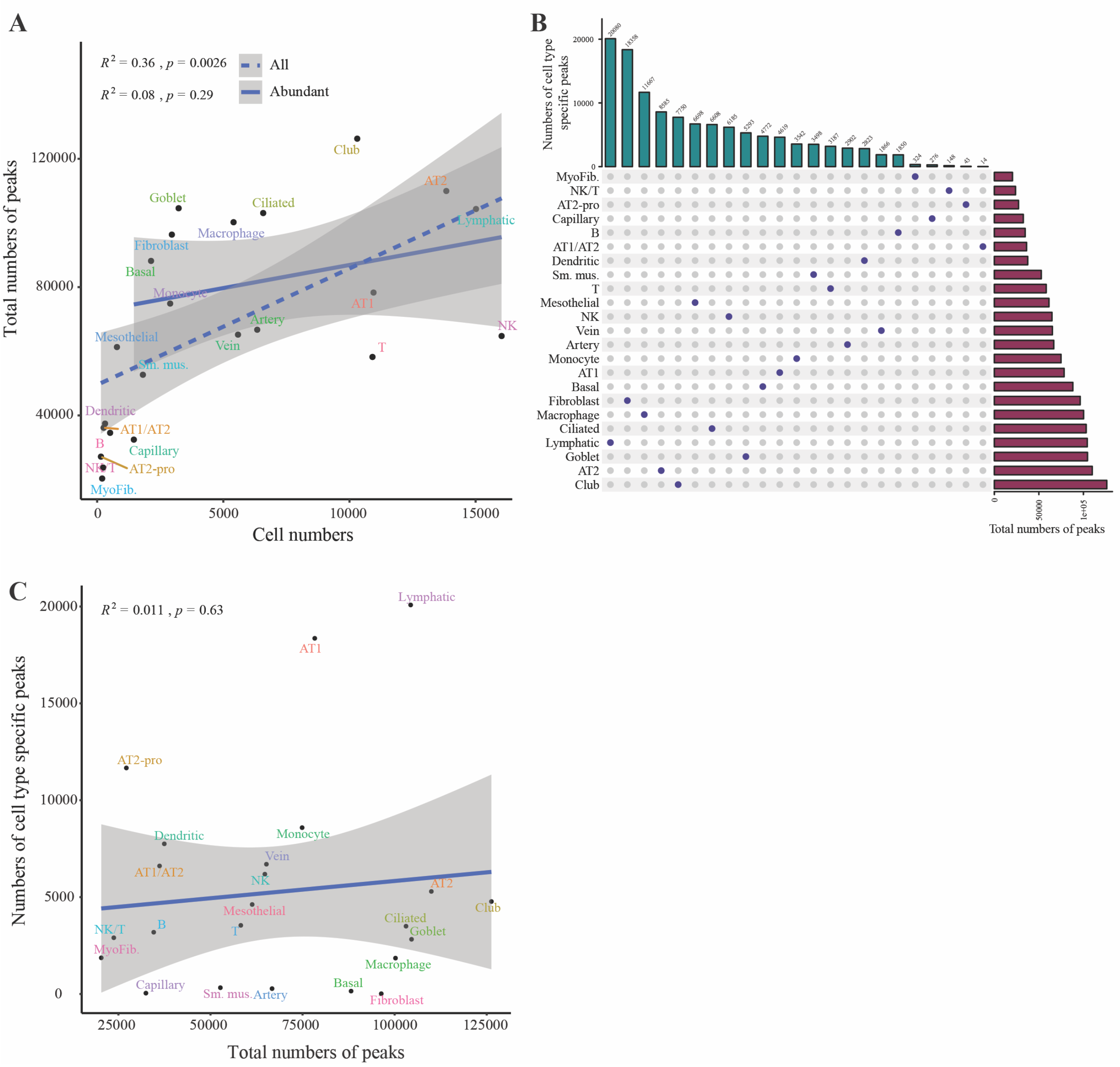

Figure S22. Association between cell numbers and peak numbers across the cell types. (A) Relationships between total numbers of peaks and cell numbers within each cell type. The dashed line shows the association among all cell types, and the solid line shows association among abundant cell types (cell proportion > 1% of total cell number). (B) Upset plot visualizes the numbers of total peaks (right purple bars) and cell type-specific peaks (top green bars). (C) Relationship between numbers of total peaks and cell type-specific peaks within each cell type. Statistical significance was tested by Pearson correlation. Source data are provided as a Source Data file.
