# Supplementary material for: Context-aware single-cell multiomics approach identifies cell-type-specific lung cancer susceptibility genes
Source: Nat Commun. 2024 Sep 12;15:7995. doi: 10.1038/s41467-024-52356-9 (PMC11392933; doi:10.1038/s41467-024-52356-9)
Supplement: Supplementary file 3 — Description of Additional Supplementary Files [file 41467_2024_52356_MOESM3_ESM.pdf]

Description of Additional Supplementary Files

Supplementary Data 1: Baseline characteristics of tumor-distant normal lung tissues collected in this study

Supplementary Data 2: Overview of the snATAC-seq and snRNA-seq QC data after Cell Ranger ARC analysis

Supplementary Data 3: Number of nuclei after filtering likely empty droplets, doublets, and low-quality nuclei

Supplementary Data 4: Cell type annotation based on canonical markers

Supplementary Data 5: Number of cell types across each sample

Supplementary Data 6: Suggestive smoking-responsive genes across cell types

Supplementary Data 7: Smoking-responsive cCREs across cell types

Supplementary Data 8: List of 51 lung cancer GWAS loci, corresponding lead SNPs, and criteria in selecting candidate causal variants

Supplementary Data 9: Complete list of candidate casual variants across lung cancer GWAS loci

Supplementary Data 10: CCV-colocalizing cCRE across lung cancer GWAS loci

Supplementary Data 11: Linking the CCV-colocalized cCREs to target genes in 6 levels

Supplementary Data 12: Level-6 linkage results between CCV-colocalized cCRE and target genes

Supplementary Data 13: All significant linkage between cCRE and gene within 1Mb

Supplementary Data 14: All significant linkage between cCRE and gene within 2Mb

Supplementary Data 15: All signifiant linkage between cCRE and gene within 5Mb

Supplementary Data 16: Linkage between CCV-colocalized cCRE and target genes in three distance windows
Supplementary Data 17: Summary of subpopulations in 16 cell types with >1,000 cells

Supplementary Data 18: Specific antibodies and conditions employed for immunohistochemical staining
