# Supplementary material for: Context-aware single-cell multiomics approach identifies cell-type-specific lung cancer susceptibility genes
Source: Nat Commun. 2024 Sep 12;15:7995. doi: 10.1038/s41467-024-52356-9 (PMC11392933; doi:10.1038/s41467-024-52356-9)
Supplement: Supplementary file 5 — Reporting Summary [file 41467_2024_52356_MOESM5_ESM.pdf]

nature portfolio

Corresponding author(s): Jiyeon Choi, Eun Young Kim, Jin Gu Lee

Last updated by author(s): 9/2/2024

Reporting Summary

Nature Portfolio wishes to improve the reproducibility of the work that we publish. This form provides structure for consistency and transparency in reporting. For further information on Nature Portfolio policies, see our Editorial Policies and the Editorial Policy Checklist.

Statistics

For all statistical analyses, confirm that the following items are present in the figure legend, table legend, main text, or Methods section.

n/a	Confirmed	
☐	☒	The exact sample size (*n*) for each experimental group/condition, given as a discrete number and unit of measurement
☐	☒	A statement on whether measurements were taken from distinct samples or whether the same sample was measured repeatedly
☐	☒	The statistical test(s) used AND whether they are one- or two-sided<br>*Only common tests should be described solely by name; describe more complex techniques in the Methods section.*
☐	☒	A description of all covariates tested
☐	☒	A description of any assumptions or corrections, such as tests of normality and adjustment for multiple comparisons
☐	☒	A full description of the statistical parameters including central tendency (e.g. means) or other basic estimates (e.g. regression coefficient) AND variation (e.g. standard deviation) or associated estimates of uncertainty (e.g. confidence intervals)
☐	☒	For null hypothesis testing, the test statistic (e.g. F, t, r) with confidence intervals, effect sizes, degrees of freedom and P value noted<br>*Give P values as exact values whenever suitable.*
☒	☐	For Bayesian analysis, information on the choice of priors and Markov chain Monte Carlo settings
☒	☐	For hierarchical and complex designs, identification of the appropriate level for tests and full reporting of outcomes
☐	☒	Estimates of effect sizes (e.g. Cohen's *d*, Pearson's *r*), indicating how they were calculated

Our web collection on statistics for biologists contains articles on many of the points above.

Software and code

Policy information about availability of computer code

Data collection	Not applicable
Data analysis	Cell Ranger ARC (v.2.0.1), DropletQC (v.0.9), R (v.4.1.3), Seurat (v.4.0.6), Scrublet, Harmony (v.1.2.0), Signac(v1.12.0), ChIPseeker, DESeq2 (v1.41.1), CellChat (version 1.6.0), SCAVENGE, ArchR, motifbreakR, Cicero. The code for reanalyzing the data reported in this paper was deposited with detailed instruction on Github via the following link: https://github.com/pumclyy/HLISCA and Zenodo: (https://doi.org/10.5281/zenodo.13306592)

For manuscripts utilizing custom algorithms or software that are central to the research but not yet described in published literature, software must be made available to editors and reviewers. We strongly encourage code deposition in a community repository (e.g. GitHub). See the Nature Portfolio guidelines for submitting code & software for further information.

Data

Policy information about availability of data

All manuscripts must include a data availability statement. This statement should provide the following information, where applicable:

- Accession codes, unique identifiers, or web links for publicly available datasets
- A description of any restrictions on data availability
- For clinical datasets or third party data, please ensure that the statement adheres to our policy

All single-cell sequencing raw (fastq or bam) and pre-processed data (h5 and Seurat object) are deposited in the Gene Expression Omnibus (GEO) database under the accession GSE241468

Research involving human participants, their data, or biological material

Policy information about studies with human participants or human data. See also policy information about sex, gender (identity/presentation), and sexual orientation and race, ethnicity and racism.

Reporting on sex and gender	We collected equal numbers of tissue samples from ever- and never- smokers while matching age and self-reported sex between two groups. Sex was used as a covariate when performing differential expression analysis between ever- and never-smoker groups.
Reporting on race, ethnicity, or other socially relevant groupings	All tissue samples were collected from ethnically Korean (self-defined) individuals through a hospital in South Korea
Population characteristics	All participants were lung adenocarcinoma patients undergoing curative lobectomy (stage I, II, and III). Their smoking status and history were collected and used for smoking-related gene expression analyses. Their ages were used for balancing the groups but not used as covariates.
Recruitment	Patients undergoing a lobectomy for lung adenocarcinoma were recruited by providing consent for the research. Patients are provided a thorough in-person explanation of the research purposes and processes. Patients are not supposed to give consent on the spot when they first learn about potential research participation and are given as much time that they needed between obtaining information and giving consent. Human-derived materials were voluntarily obtained through informed consent for human-derived material research (HMR ICF), by the Enforcement Rule of Bioethics and Safety Act of the Republic of Korea. Human-derived materials refer to lung tissues and blood collected from humans, as well as chromosomes, DNA, RNA, proteins, and other substances isolated from these samples. All samples were collected primarily to study risk factors and prognoses of respiratory diseases. The donors consented to the secondary use of their samples for comprehensive research purposes, including providing data containing personally identifiable information such as sex, age and the medical center.
Ethics oversight	Patient tissues were obtained under a protocol approved by Yonsei University Health System, Severance Hospital, Institutional Review Board (IRB 4-2019-0447, 4-2022-0706), and informed consent was obtained from each patient prior to surgery.

Note that full information on the approval of the study protocol must also be provided in the manuscript.

Field-specific reporting

Please select the one below that is the best fit for your research. If you are not sure, read the appropriate sections before making your selection.

☒ Life sciences ☐ Behavioural & social sciences ☐ Ecological, evolutionary & environmental sciences

For a reference copy of the document with all sections, see nature.com/documents/nr-reporting-summary-flat.pdf

Life sciences study design

All studies must disclose on these points even when the disclosure is negative.

Sample size	The sample size of comparing 8 vs. 8 for assessing smoking effect was determined based on a recent scRNA-seq study that has successfully detected differential gene expression between smokers (n = 6) and non-smokers (n = 6) in human tracheal epithelium
Data exclusions	Single-cell data was filtered to exclude low quality cells as described in Quality control and filtering and Clustering and cell-type annotation section of Methods
Replication	Cell-cell interaction analysis were replicated in an external dataset (62 normal human lung samples from Human Lung Cell Atlas (HLCA) core dataset based on the availability of the smoking status information)
Randomization	Not applicable (groups were defined based on the self-reported smoking status)

Blinding	Smoking groups were blinded during the tissue sample dissociation step

Reporting for specific materials, systems and methods

We require information from authors about some types of materials, experimental systems and methods used in many studies. Here, indicate whether each material, system or method listed is relevant to your study. If you are not sure if a list item applies to your research, read the appropriate section before selecting a response.

Materials & experimental systems

n/a	Involved in the study
☐	☒ Antibodies
☐	☒ Eukaryotic cell lines
☒	☐ Palaeontology and archaeology
☒	☐ Animals and other organisms
☒	☐ Clinical data
☒	☐ Dual use research of concern
☒	☐ Plants

Methods

n/a	Involved in the study
☒	☐ ChIP-seq
☐	☒ Flow cytometry
☒	☐ MRI-based neuroimaging

Antibodies

Antibodies used	FITC anti-human CD45 (HI30; 304006, BioLegend), APC anti-human CD31 (WM59; 303116, BioLegend), anti-human CD326 1B7 PE-CYN7(25-9326-42, ThermoFisher Scientific), Cytokeratin 17 (E-4, sc-393002, Santa Cruz Biotechnology), Ki67 (ab238020, Abcam), Surfactant protein D/SP-D (ab220423, Abcam), TERT (A-6, sc-393013, Santa Cruz Biotechnology)
Validation	All the antibodies were validated by the manufacturers.

Eukaryotic cell lines

Policy information about cell lines and Sex and Gender in Research

Cell line source(s)	A549 cells were originally isolated from the lung tumor tissue of a 58-year old white male suffering from lung cancer.
Authentication	Routine STR profiling was carried out to ensure the authenticity of A549 lung cancer cells.
Mycoplasma contamination	A549 cells used in the study were free from mycoplasma contamination as confirmed by routine mycoplasma detection assays.
Commonly misidentified lines (See ICLAC register)	A549 lung cancer cells are not among the list of commonly misidentified cell lines (latest version 13).

Plants

Seed stocks	Not applicable
Novel plant genotypes	Not applicable
Authentication	Not applicable

Flow Cytometry

Plots

Confirm that:

☒ The axis labels state the marker and fluorochrome used (e.g. CD4-FITC).

☒ The axis scales are clearly visible. Include numbers along axes only for bottom left plot of group (a 'group' is an analysis of identical markers).

☒ All plots are contour plots with outliers or pseudocolor plots.

☒ A numerical value for number of cells or percentage (with statistics) is provided.

Methodology

Sample preparation	Dissociated single-cell suspensions were thawed and filtered using a 70 µm cell strainer (Miltenyi Biotec) to remove debris. The filtered cells were labelled with cell viability marker (DAPI) and antibody markers of EPCAM (CD326), CD31, and CD45.
Instrument	BD FACSAria Fusion Flow Cytometer (BD Biosciences)
Software	BD FACSDiva Software
Cell population abundance	To enrich epithelial cells, which are considered to have key roles in lung cancer etiology, we collected all "epithelial" cells from EPCAM+CD45- gates and balanced the ratios roughly to 6:3:1 ("epithelial": "immune": "endothelial or stromal").
Gating strategy	Live single-cells (DAPI-negative) were sorted based on three gates: EPCAM+CD45- (designated "epithelial"), EPCAM-CD45+ (designated "immune"), and EPCAM-CD45- (designed "endothelial or stromal").

☒ Tick this box to confirm that a figure exemplifying the gating strategy is provided in the Supplementary Information.
